# Patients With Skin Cancer Prefer to Participate in Procedure Cost Discussions: A Cross-Sectional Survey

**DOI:** 10.1155/drp/1660527

**Published:** 2025-09-25

**Authors:** Ritika Bhandari, Laura Rezac, Ross L. Pearlman, Vinayak K. Nahar, William H. Black

**Affiliations:** Department of Dermatology, University of Mississippi Medical Center, Jackson, Mississippi, USA

**Keywords:** cost, Mohs micrographic surgery, patient discussion, skin cancer

## Abstract

For a given skin cancer, a number of treatment options are often available. The decision of which method to use is usually made by the treating physician. Despite significant changes to the healthcare system of the United States over the past 10 years, healthcare costs continue to rise. These costs often affect patients in the form of higher deductibles, copays, and insurance premiums. The goal of this study was to determine patient attitudes regarding discussion of cost of skin cancer removal procedures and repairs. A 12-question survey was administered to 100 patients presenting for treatment of a skin cancer at an academic center. The first six questions addressed the importance the patient placed on treatment cost and related discussions, and the final six questions addressed repair cost. Greater than two-thirds of respondents felt that cost of both treatment (76%) and repair (67%) is somewhat or very important. Most patients reported that the cost of skin cancer treatment (56%) and repair (54%) should be considered by their surgeon. Furthermore, a majority of participants felt that cost differences should be discussed prior to treatment (67%) or repair (67%). Most respondents believed that cost discussion prior to treatment (64%) and repair (67%) would not affect their level of procedural anxiety. In conclusion, patients value cost discussions for treatment and repair of skin cancer. Surgeons should consider discussing these issues with patients in the appropriate clinical setting.

## 1. Introduction

Patient knowledge of options and associated costs prior to consent for procedures is often limited and may vary significantly. A 2003 study found that only 15% of patients had discussed out-of-pocket costs with their physician prior to treatment [1], while a 2012 study of oncology patients reported that most preferred cost discussions with their physicians [2]. More recently, a 2022 national cross-sectional electronic survey by Lam and colleagues [3] showed that while 62% of cancer or autoimmune patients desired cost discussions, only 32% reported that such discussions occurred. Byzantine insurance policies, contracts between various healthcare organizations and payers, and managed care organizations may also complicate cost considerations for patients [4].

Patients with malignant tumors of the skin are frequently referred to dermatologic surgeons for definitive treatment options. The approach to treating a particular skin cancer is typically determined by the consulting surgeon with input from the patient. Different categories of treatment include surgical management, destructive modalities, and nonsurgical therapies including topical therapy and radiation [5]. Each option offers different clinical utility and therapeutic efficacy that can be balanced with cost and patient desire.

Surgical management techniques, regarded as the most effective methods of treatment, include Mohs micrographic surgery (MMS) and standard surgical excision (SSE) with postoperative pathologic assessment. In many cases, both techniques may be appropriate. Although more expensive than SSE, MMS has been found to be a cost-effective procedure with very high initial cure rates, low rates of recurrence, and rare associated facility fees [6]. Also impacting the total cost of treatment is the type of repair, the choice of which can vary greatly from surgeon to surgeon, and in terms of expense. In most cases, the surgeon decides how much to involve patients in these decisions regarding treatment [7, 8]. We are not aware of previous studies that address patient inclusion in these discussions as they relate to surgical treatment of skin cancer and cost. Thus, the aim of this study was to examine general patient attitudes regarding discussions of cost of skin cancer treatment and repairs.

## 2. Methods

This study was approved by the Institutional Review Board at the University of Mississippi Medical Center (IRB #2017-0242). A paper questionnaire was offered to patients over 18 years of age after MMS at the University of Mississippi Medical Center's Skin Cancer Center. The first 100 patients, invited over the course of approximately 1 month, who consented to participate were given a 12-question survey. Collected patient information was limited to de-identified age and sex. Questions addressed the following issues regarding both treatment and repair: the importance of cost to the patient and the provider, patient-provider discussion of cost, patient perceptions of providers as it relates to such discussions, anticipated patient anxiety regarding cost information, and previous experience with such discussions. Similar questions regarding the cost of treatment and cost of repair were asked. For treatment questions, patients were first educated that several treatment options often exist for a given cancer. For repair questions, it was explained that multiple modalities usually exist including allowing the wound to heal on its own, and that costs may vary greatly.

A preprint of this manuscript has previously been published on Research Square [9].

## 3. Results

A total of 100 participants, including both melanoma and keratinocyte carcinoma (KC) patients, completed the survey. Of these, 68% were male, and the mean age for all participants was 65.89 years (standard deviation ± 10.92). In general, questions regarding treatment costs (Table 1) were similar to those regarding repair costs (Table 2). Greater than two-thirds felt that cost of treatment/repair (76%, 67%) is somewhat or very important. A small majority (56%, 54%) of participants felt that cost differences should be considered by their doctor. Roughly two-thirds (67%, 63%) agreed that cost differences should be discussed prior to treatment. Most (72%, 70%) would have no change in opinion of their doctor based on such discussions. No participants felt they would have a negative opinion of their doctor if cost was emphasized. More (20%, 18%) felt their anxiety would be decreased by discussion of cost than felt it would be increased (6%, 7%), although most (64%, 67%) felt it would not change their anxiety. Of those treated previously, the vast majority (76%, 82%) reported that cost had never been discussed with them.

## 4. Discussion

Our results suggest that when multiple options exist for skin cancer removal and repair, most patients find cost important and prefer physicians to consider differences in procedure costs. Most patients also prefer to have options discussed with them, though most with a history of procedures have not had that experience. These findings are in line with Lam et al., who found that although 62% of cancer or autoimmune patients wanted cost discussions, only 32% reported having such discussions [3]. Although we considered that a “choice paralysis” may inhibit patients when asked to contribute to treatment decisions, less than 10% felt that this responsibility would increase anxiety. In our practice, cost typically has been discussed only with self-paying patients. Discussions are most often in general terms, as the physician does not usually know the exact cost difference between two options. For instance, the cost of MMS cannot be determined prior to the procedure because the number of Mohs stages and type and complexity of repair contribute to the overall charge. Arguably the most significant obstacle to cost discussion relates to a patient's out-of-pocket cost depending on their insurance plan.

The complexity of individual insurance coverage, deductibles, etc. is too great to make discussion of specific costs practical on a regular basis. Therefore, a general discussion based on estimates would be the most useful. Development of general cost estimates for patients would depend on the unique practice patterns of each surgeon along with the health insurance environment in the surgeon's region. For example, patients that express cost sensitivity prior to repair could be offered a linear repair rather than a flap where appropriate. Each option's risks, benefits, and general costs would be discussed with the patient in a shared decision-making model. Preliminary data suggest that these models have the ability to reduce costs for patients while also preserving quality of services and patient satisfaction [10]. Due to limited time during patient encounters, office staff rather than clinical staff could be incorporated into this discussion. Cost discussions should enhance the informed consent process, not provide financial planning services to the patient or guide therapy.

This study was distributed to patients after undergoing MMS and not offered to patients undergoing skin cancer treatment with other modalities, and this method of distribution represents an important limitation of the study. Additionally, cost sensitivity may differ from state to state and among different practice settings due to the complexities of payor coverage. Future studies should experiment with development and implementation of cost discussions as part of the informed consent process when treating skin cancers.

## Figures and Tables

**Table 1:** Survey responses of the participants regarding treatment of skin cancer.

Survey questions	Respondents, no. (%)
1. With regard to treatment options for your skin cancer, how important is the cost to you?	
Very important/	25 (25)
Somewhat important	51 (51)
Not important	20 (20)
I don't know	4 (4)
2. With regard to treatment of your skin cancer, should the difference in cost of treatments be considered by your doctor?	
Yes	56 (56)
No	32 (32)
I don't know	12 (12)
3. With regard to treatment of your skin cancer, should your doctor discuss with you the difference in costs prior to treating?	
Yes	67 (67)
No	24 (24)
I don't know	9 (9)
4. If your doctor emphasized the cost of treatment options in your pretreatment discussion, would this change your opinion of your doctor?	
He/she is a better doctor	25 (25)
He/she is a worse doctor	0 (0)
No change in opinion	72 (72)
I don't know	3 (3)
5. If you were asked to make a treatment choice, after being given several options and cost information, how would that affect your anxiety about the process?	
More anxiety	6 (6)
Less anxiety	20 (20)
No change in anxiety	64 (64)
I don't know	10 (10)
6. If you have been treated for skin cancer previously, how often has the cost of treatment been discussed prior to treatment?	
Never	56 (56)
Seldom	12 (12)
Sometimes	3 (3)
Often	1 (1)
I don't know	2 (2)
I've never been treated	26 (26)

**Table 2:** Survey responses of the participants regarding repair after treatment of skin cancer.

Survey questions	Respondents, no. (%)
1. With regard to repair options (suturing), how important is the cost to you?	
Very important/	14 (14)
Somewhat important	52 (52)
Not important	29 (29)
I don't know	5 (5)
2. With regard to repair of a wound after excision (suturing), should the difference in cost of repairs be considered by your doctor?	
Yes	54 (54)
No	30 (30)
I don't know	16 (16)
3. With regard to repair of a wound after excision (suturing), should your doctor discuss with you the difference in costs prior to treating?	
Yes	63 (63)
No	26 (26)
I don't know	11 (11)
4. If your doctor emphasized the cost of repair options in your pretreatment discussion, how would that change your opinion of your doctor?	
He/she is a better doctor	26 (26)
He/she is a worse doctor	0 (0)
No change in opinion	70 (70)
I don't know	4 (4)
5. If you were asked to make a treatment choice, after being given several options and cost information, how would that affect your anxiety about the process?	
More anxiety	7 (7)
Less anxiety	18 (18)
No change in anxiety	67 (67)
I don't know	8 (8)
6. If you were asked to make a repair choice, after being given several options and cost information, how would that affect your anxiety about the process?	
Never	58 (58)
Seldom	8 (8)
Sometimes	4 (4)
Often	0 (0)
I don't know	1 (1)
I've never been treated	29 (29)

## Data Availability

The data that support the findings of this study are available on request from the corresponding author. The data are not publicly available due to privacy or ethical restrictions.

## References

[1] Alexander G. C., Casalino L. P., Meltzer D. O. (2003). Patient-Physician Communication About Out-of-Pocket Costs. *JAMA*.

[2] Bullock A. J., Hofstatter E. W., Yushak M. L., Buss M. K. (2012). Understanding Patients’ Attitudes Toward Communication About the Cost of Cancer Care. *Journal of Oncology Practice*.

[3] Lam A. B., Nipp R. D., Hasler J. S. (2024). National Survey of Patient Perspectives on Cost Discussions Among Recipients of Copay Assistance. *The Oncologist*.

[4] Benavidez G., Frakt A. (2019). Price Transparency in Health Care Has Been Disappointing, But it Doesn’t Have to be. *JAMA*.

[5] Rogers H. W., Coldiron B. M. (2009). A Relative Value Unit-Based Cost Comparison of Treatment Modalities for Nonmelanoma Skin Cancer: Effect of the Loss of the Mohs Multiple Surgery Reduction Exemption. *Journal of the American Academy of Dermatology*.

[6] Tierney E. P., Hanke C. W. (2009). Cost Effectiveness of Mohs Micrographic Surgery: Review of the Literature. *Journal of Drugs in Dermatology*.

[7] Hardee J. T., Platt F. W., Kasper I. K. (2005). Discussing Health Care Costs With Patients. *Journal of General Internal Medicine*.

[8] Henrikson N. B., Chang E., Ulrich K., King D., Anderson M. L. (2017). Communication With Physicians About Health Care Costs: Survey of an Insured Population. *The Permanente Journal*.

[9] Bhandari R., Rezac L., Pearlman R., Nahar V., Black W. (2022). Patients With Skin Cancer Prefer to Participate in Procedure Cost Discussions: A Cross-Sectional Survey. *Preprint*.

[10] Veroff D., Marr A., Wennberg D. E. (2013). Enhanced Support for Shared Decision Making Reduced Costs of Care for Patients With Preference-Sensitive Conditions. *Health Affairs*.

